# Human monocyte-derived type 1 and 2 macrophages recognize Ara h 1, a major peanut allergen, by different mechanisms

**DOI:** 10.1038/s41598-021-89402-1

**Published:** 2021-05-12

**Authors:** Maren Krause, Peter Crauwels, Frank Blanco-Pérez, Martin Globisch, Andrea Wangorsch, Thomas Henle, Jonas Lidholm, Ger van Zandbergen, Stefan Vieths, Stephan Scheurer, Masako Toda

**Affiliations:** 1 VPr1 Research Group “Molecular Allergology“, Paul-Ehrlich-Institut, Langen, Germany; 2 Division of Immunology, Paul-Ehrlich-Institut, Langen, Germany; 3 Institute of Microbiology and Biotechnology, University of Ulm, Ulm, Germany; 4 Institute of Food Chemistry, Technische Universität Dresden, Dresden, Germany; 5 Thermo Fisher Scientific, Uppsala, Sweden; 6 Institute for Immunology, University Medical Center, Johannes Gutenberg University Mainz, Mainz, Germany; 7 Research Center for Immunotherapy (FZI), University Medical Center, Johannes Gutenberg-University Mainz, Mainz, Germany; 8 Laboratory of Food and Biomolecular Science, Graduate School of Agricultural Science, Tohoku University, Sendai, Japan

**Keywords:** Immunology, Molecular biology

## Abstract

Evidence has suggested that major peanut allergen Ara h 1 activates dendritic cells (DCs) via interaction with DC-SIGN (dendritic cell-specific intercellular adhesion molecule-3-grabbing non-integrin), a C-type lectin receptor, and contributes to development of peanut allergy. Since macrophages, as well as DCs, play a crucial role in innate immunity, we investigated whether natural Ara h 1 (nAra h 1) activates two different subsets of macrophages, human monocyte derived macrophage type 1 (hMDM1: pro-inflammatory model) and type 2 (hMDM2: anti-inflammatory model). hMDM1 and hMDM2 predominantly produced pro-inflammatory cytokines (IL-6 and TNF-α) and an anti-inflammatory cytokine (IL-10) in response to nAra h 1, respectively. hMDM2 took up nAra h 1 and expressed DC-SIGN at higher levels than hMDM1. However, small interfering RNA knockdown of DC-SIGN did not suppress nAra h 1 uptake and nAra h 1-mediated cytokine production in hMDM2. Inhibitors of scavenger receptor class A type I (SR-AI) suppressed the response of hMDM2, but not of hMDM1, suggesting that SR-AI is a major receptor in hMDM2 for nAra h 1 recognition and internalization. nAra h 1 appears to exert stimulatory capacity on DC and macrophages via different receptors. This study advances our understanding how a major peanut allergen interacts with innate immunity.

## Introduction

Accumulated evidence indicates that some proteins are capable of stimulating innate immunity. For allergic diseases, it is thought that the stimulatory capacity of allergens in innate immunity contributes to the initiation of inflammation and development of Th2 immune responses^1,2^. These capacity of allergens may be mediated by intrinsic properties e.g. their protease activity damaging integrity of epithelial layers^2–4^, feature to mimic accessory proteins (e.g., Myeloid differentiation-2 (MD2)) binding capacity of allergens with lipids triggering Toll-like receptor (TLR) 4-mediated inflammatory responses^3–6^, or the presence of carbohydrate residues stimulating antigen presenting cells (APC) via interaction of pattern recognition receptors (PPRs)^7–10^. Moreover, the interaction of allergens with APCs in innate immunity could also be a factor influencing the efficacy of allergen-specific immunotherapy^11,12^. APCs orchestrate immune responses by linking innate and adaptive immunity and contribute to induction of desensitization or peripheral tolerance^13,14^.

Peanuts are one of the most common allergenic foods with the capacity of causing a life-threatening allergic reaction^15^. Ara h 1 is a major allergen, potentially contributing to peanut-induced anaphylaxis and belongs to the vicilin (7S) family of seed storage proteins^16^. A previous study suggested that natural Ara h 1 (nAra h 1) activates human monocyte derived dendritic cells (hMDDC) by binding DC-SIGN (Dendritic Cell-Specific Intercellular adhesion molecule-3-Grabbing Non-integrin) and thereby induces Th2 skewing of naive T cells^17^. nAra h 1 carries high mannose (Man5GlcNAc2 and Man6GlcNAc2) and nonfucosylated complex N-glycans (Man4XylGlcNAc2 and Man3XylGlcNAc2), which would be most likely recognized by PPRs including DC-SIGN^17,18^. PPRs include TLRs, Nod-like receptors (NLRs), C-type lectin receptors (CLRs) and scavenger receptors (SRs)^19–21^. PRRs are primarily present in professional APC such as dendritic cells (DC) and macrophages. The expression profiles of PPRs are different between phenotypes of DC and macrophages^21,22^. However, in comparison to DC, the engagement of macrophages in recognition of allergens has not been investigated in detail.

In the present study, we aimed to investigate the interaction between nAra h 1 and human monocyte-derived macrophages. Depending on the local microenvironment, macrophages are polarized into either classically activated (pro-inflammatory M1) or alternatively activated (anti-inflammatory M2) phenotypes^12,22–24^. In current standard, M1 and M2 macrophage polarization is defined as stimulating bone marrow-derived murine macrophages with LPS plus IFN-γ or IL-4 in vitro^24^*.* A previous study showed that human monocyte derived macrophage type 1 (hMDM1) and type 2 (hMDM2) exhibits similar profiles of receptor expression and function with M1 and M2 macrophages, respectively^25^. hMDM1 were characterized by their fried-egg shaped morphology and CD14^+^ MHCII^+^ CD163^-^ phenotype, whereas anti-inflammatory hMDM2 were characterized by CD14^+^ MHCII^+^ CD163^+^ with more elongated cell bodies^25^. Using such hMDM1 and hMDM2, we found that (i) nAra h 1 activates both macrophage phenotypes but via different receptors, (ii) DC-SIGN is likely not a major receptor for nAra h 1-mediated activation in the both phenotypes, and (iii) macrophage scavenger receptor class A type I (SR-AI) is involved in a recognition of Ara h 1 by hMDM2.

## Materials and methods

### Preparation of Ara h 1 samples

Natural Ara h 1 was isolated from unroasted peanuts following a protocol established by Maleki et al.^26^. SDS-PAGE profile and blotting analysis of nAra h 1 is indicated in Fig. S1. Recombinant mature Ara h 1.0101 (rAra h 1, Acc. No. P43238, excluding the 25-residue signal peptide) was expressed as a C-terminally hexahistidine tagged protein in *E. coli* BL21-AI. The protein was purified from the soluble cell fraction by immobilized metal ion affinity chromatography (IMAC) and anion exchange chromatography, followed by buffer exchange to 20 mM MOPS, 0.15 M NaCl, pH 7.6. Folding of recombinant protein was confirmed by circular dichroism analysis (J-810S spectropolarimeter (Jasco) (Fig. S2). The protein concentration was determined from absorbance at 280 nm using an extinction coefficient of 0.57 mg/mL.

### Generation of hMDM1 and hMDM2

Buffy coats obtained from anonymous healthy blood donors were purchased from the German Red Cross Blood Donor Service Baden-Württemberg & Hessen. Human peripheral mononuclear cells were isolated from the buffy coats by passage over a Leukocyte Separation Medium (PAA Laboratories GmbH) gradient. Monocytes were isolated by exploiting their ability to adhere to plastic, or by CD14 positive selection (Miltenyi Biotec GmbH). Monocytes were then differentiated into hMDM1 and hMDM2 macrophages by the addition of 10 ng/mL recombinant human GM-CSF (R&D Systems) or recombinant human M-CSF for 5 to 7 days, respectively^25^.

### Assessment of macrophage activation

hMDM1 or hMDM2 (1 × 10^6^ cells/mL) were stimulated with 10 or 50 µg/mL nAra h 1, or with 0.5 µg/mL lipopolysaccharide (LPS, Sigma-Aldrich) for 18 h. The concentrations of TNF-α, IL-6 and IL-10 in the culture supernatant were measured by ELISA (Peprotech). To assess cell maturation, non-stimulated, or stimulated cells were treated with human Fc block (Biolegend) and stained with phycoerythrin (PE)-conjugated rat anti-human CD40, CD80, CD86 or HLA-DR monoclonal antibody (mAb). The fluorescence intensities of the samples were assessed by flow cytometry (LSRII, BD Bioscience). Gating strategy for FACS analysis was shown in Fig. S3. Antibodies used for this experiment were purchased from Thermo Fisher Scientific.

### Assessment of macrophage’s uptake of nAra h 1 and rAra h 1 by FACS

nAra h 1 and rAra h 1 were conjugated with fluorescein isothiocyanate (FITC) using a FluoroTag FITC conjugation kit (Sigma-Aldrich), according to the manufacturer’s instruction. Cells (1.0 × 10^6^ cells/mL) were incubated for 15 min with FITC-conjugates of the samples. Subsequently, the cells were stained with PE anti-human CD11b mAb. The FITC intensity in the cell population was analyzed by flow cytometry. To inhibit possible uptake mediated by receptors, the cells were treated with following inhibitors for 30 min before the addition of FITC conjugated samples: 200 μg/mL mannan (Sigma-Aldrich), 200 μg/mL acetylated low-density lipoprotein (Ac-LDL) (Thermo Fisher Scientific), 10 μg/mL polyinosinic acid (Sigma-Aldrich) and 100 μg/mL fucoidan (Sigma-Aldrich).

### Assessment of macrophage’s uptake of nAra h 1 by confocal microscope

To verify endocytosis of nAra h 1 by receptor-mediated uptake, hMDM1 and hMDM2 were fixed with 4% paraformaldehyde solution after incubation of FITC-conjugated nAra h 1 for 15 min. The cells were then blocked with normal goat IgG, stained with anti-human EEA1 (Thermo Fisher Scientific) in combination with Cy3-conjugated goat anti-mouse IgG, F(ab')2 fragment (Jackson ImmunoResearch), and treated with 4′,6-diamidino-2-phenylindol (DAPI: Thermo Fisher Scientific). The cells were analyzed using a laser scanning microscope (LSM 510, Carl Zeiss).

### Assessment of receptor expression

hMDM1 and hMDM2 were treated with human Fc block and stained with phycoerythrin (PE)-conjugated anti-human SR-AI (CD204), or anti-human DC-SIGN (CD209) mAb and Allophycocyanin (APC)-conjugated anti-human CD11b mAb. Antibodies used for this experiment were purchased from Thermo Fisher Scientific. The fluorescence intensities of the samples were assessed by flow cytometry.

### siRNA gene silencing

hMDM2 (1 × 10^6^ cells/mL) were transfected with DC-SIGN–specific, or nontargeting control siRNA pools (Allstar negative control siRNA) from Qiagen using Stemfect RNA transfection kit (Tebu-Bio) following the manufacturer's guidelines. The expression of DC-SIGN on the cell surface was analyzed by FACS. After 96 h, cellular uptake and activation experiments were performed as described above.

### Statistical analysis

Significant differences between mean values were assessed using paired T-test and Graph Pad Prism (GraphPad Software).

## Results

### Natural Ara h 1 activates hMDM1 and hMDM2

**Figure 1:**
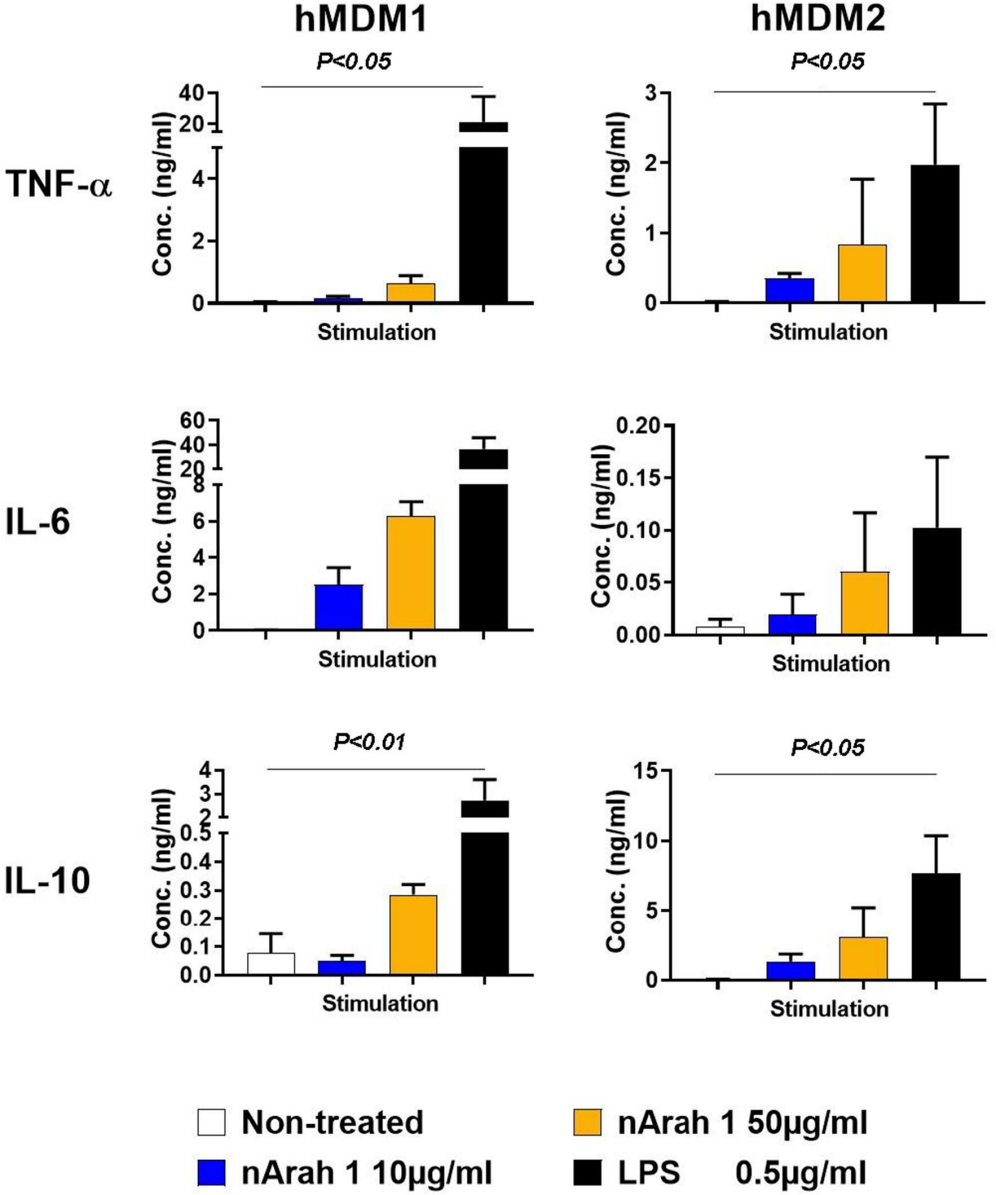
nAra h 1 induced cytokine production of hMDM1 and hMDM2. hMDM1 and hMDM2 were stimulated with 10 μg/ml (blue bar), or 50 μg/ml (orange bar) of nAra h 1, or 0.5 μg /ml of LPS (black bar), or non-treated (white bar) for 18 h. After the stimulation, concentrations of TNF-ɑ, IL-6, and IL-10 in the cell culture supernatants were measured by ELISA. The data represent three independent experiments using five donors in total.

**Figure 2:**
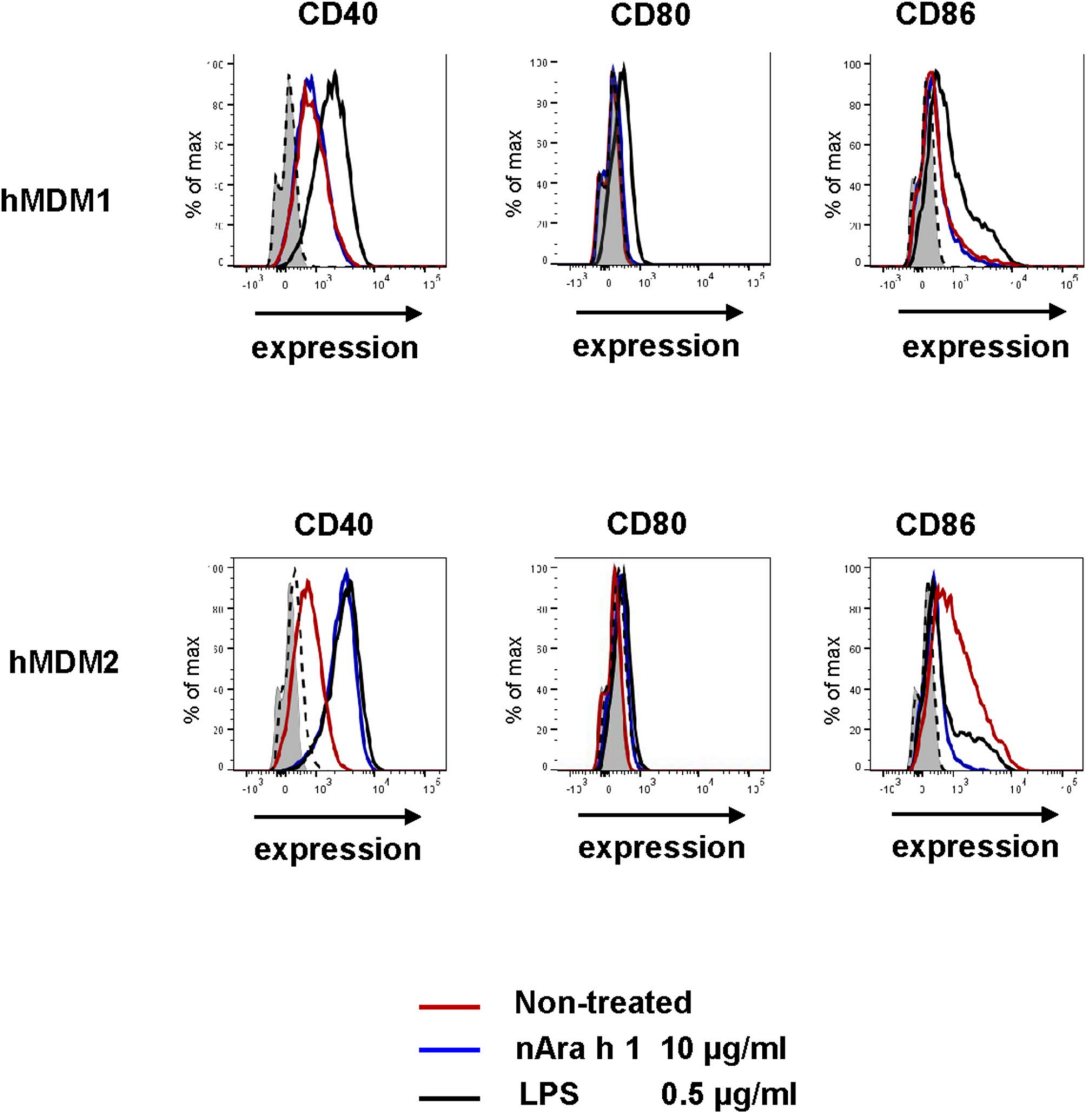
nAra h 1 induced activation of hMDM1 and hMDM2. hMDM1 and hMDM2 were stimulated with 10 μg/ml of Ara h 1 (blue line), or 0.5 μg /ml of LPS (black line), or non-treated (red line) for 18 h. After the stimulation, cell surface expression of CD40, CD80 and CD86 were measured by FACS. Grey area: cells without stimulation and staining with mAb, dashed line: cells stimulated with nAra h 1 and stained with an isotype control antibody. The data represent two independent experiments using three donors in total.

To investigate how nAra h 1 interact with human macrophages, we stimulated hMDM1 and hMDM2 with the peanut allergen isolated from raw peanuts, or LPS, a control of positive stimuli. In cell culture supernatant of nAra h 1-, or LPS-stimulated cells, TNF-α, IL-6 and IL-10 were detected (Fig. 1). hMDM1 tended to produce higher amounts of IL-6 but lower amounts of TNF-α and IL-10 than hMDM2 in response to nAra h 1. In addition, we observed enhanced expression of CD40, CD80 and CD86, which are activation markers, on the cell surface of nAra h 1-stimulated M1 macrophages, although the enhancement effect of nAra h 1 on CD40 expression was low (Fig. 2). Enhanced expression of CD40 and CD80, but reduced expression of CD86 were observed on the cell surface of nAra h 1-stimulated hMDM2. Reduced expression of CD86 was also observed in LPS-stimulated hMDM2, which was likely due to overstimulation of the cells^27^. The activation of macrophages by nAra h 1 was not due to contamination of LPS in the allergen sample, since HEK293 clones transfected with human TLR4 did not react to nAra h 1 (Fig. S4). The results indicate that nAra h 1 activates both hMDM1 and hMDM2, and the activation is not due to TLR4 engagement.

**Figure 3:**
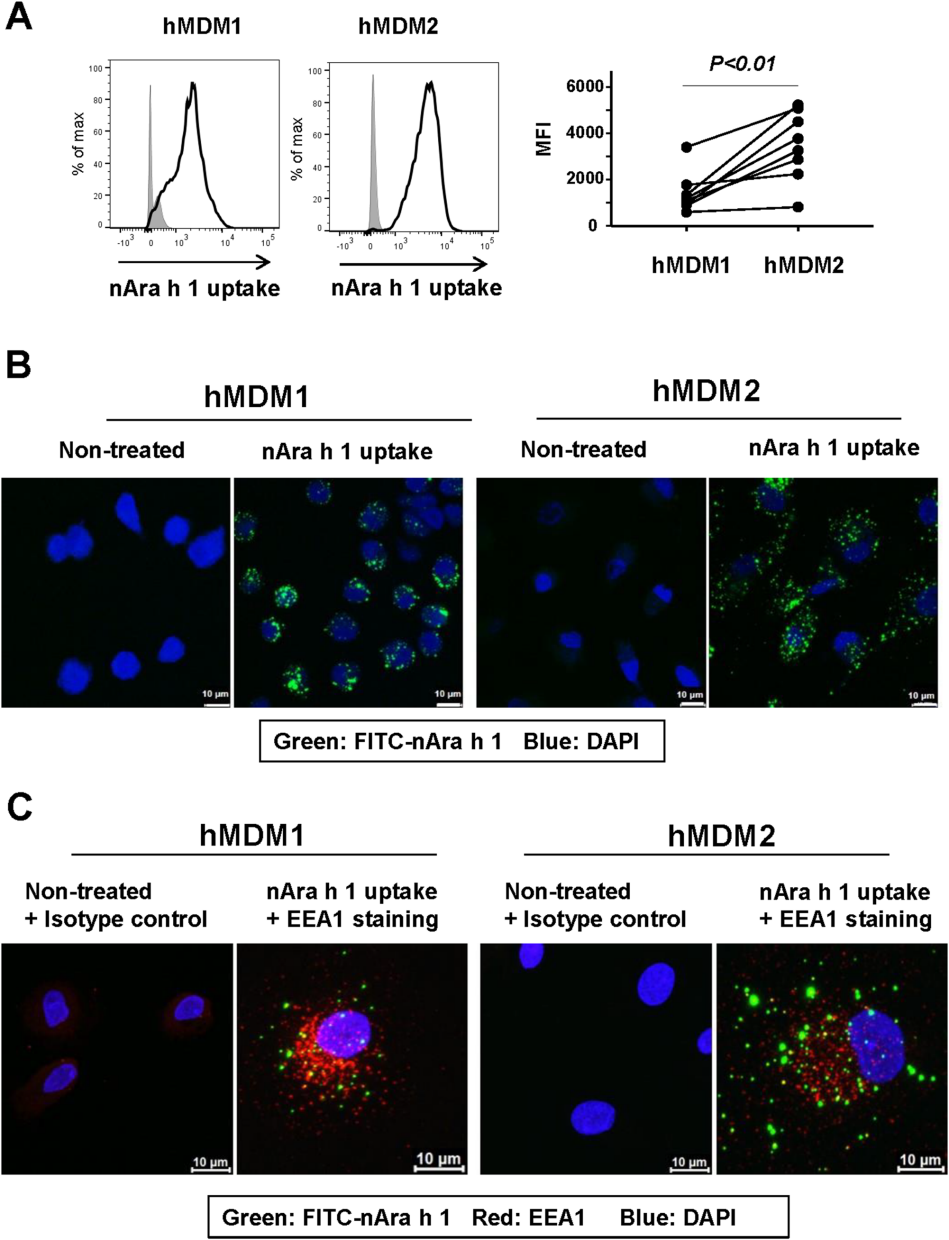
nAra h 1 was endocytosed by hMDM1 and hMDM2. hMDM1 and hMDM2 were treated with 10 µg/ml of FITC conjugated nAra h 1 (FITC-nAra h 1) for 15 min. (**A**) The uptake levels of FITC-nAra h 1 by the cells were measured by FACS. Grey area: cells without treatment, solid line: cells treated with FITC-nAra h 1. Histograms represent four independent experiments using eight donors in total. MFI: Mean fluorescence intensity in cells treated with FITC-nAra h 1. Each set of symbols connected by a line represents data from cells of an individual donor. (**B**) After the treatment, the cells were fixed with 4% paraformaldehyde and stained with DAPI. The uptake of FITC-nAra h 1 was verified by confocal microscopy. The data represent two independent experiments using four donors in total. (**C**) After the treatment and fixation, the cells were stained with anti-EEA1 mAb and DAPI. The cell images in Z-stack were obtained by confocal microscopy. Green, red, and blue colors indicate FITC-nAra h 1, EAA1 and DAPI, respectively. The data represent two independent experiments using four donors in total.

Next, we assessed levels of nAra h 1 uptake by macrophages. The cells were incubated with FITC-conjugated nAra h 1. The FITC intensity of macrophages was analyzed by flow cytometry as a measure of allergen uptake. We observed that hMDM2 took up Ara h 1 at higher levels than hMDM1 (Fig. 3A). Subsequent confocal microscopy analyses showed that the most of the nAra h 1 was not merely attached on the cell surface of macrophages, but was endocytosed into the cells, although it was not colocalized with early endosome antigen 1 (EAA1) (Figs. 3B,C, S5, S6) The result suggests that nAra h 1 was endocytosed but not into early endosomes.

To investigate whether the uptake of nAra h 1 by the cells was due to the presence of glycan in the allergen, we assessed the uptake of rAra h 1 without glycan, which was prepared in an *E. coli* expression system. The uptake of rAra h 1 by hMDM1 and hMDM2 was at marginal levels (Fig. S7). The results indicated that glycan is a key component in the interaction of nAra h 1 with macrophages.

### DC-SIGN is not involved in hMDM1 and hMDM2 activation by natural Ara h 1

**Figure 4:**
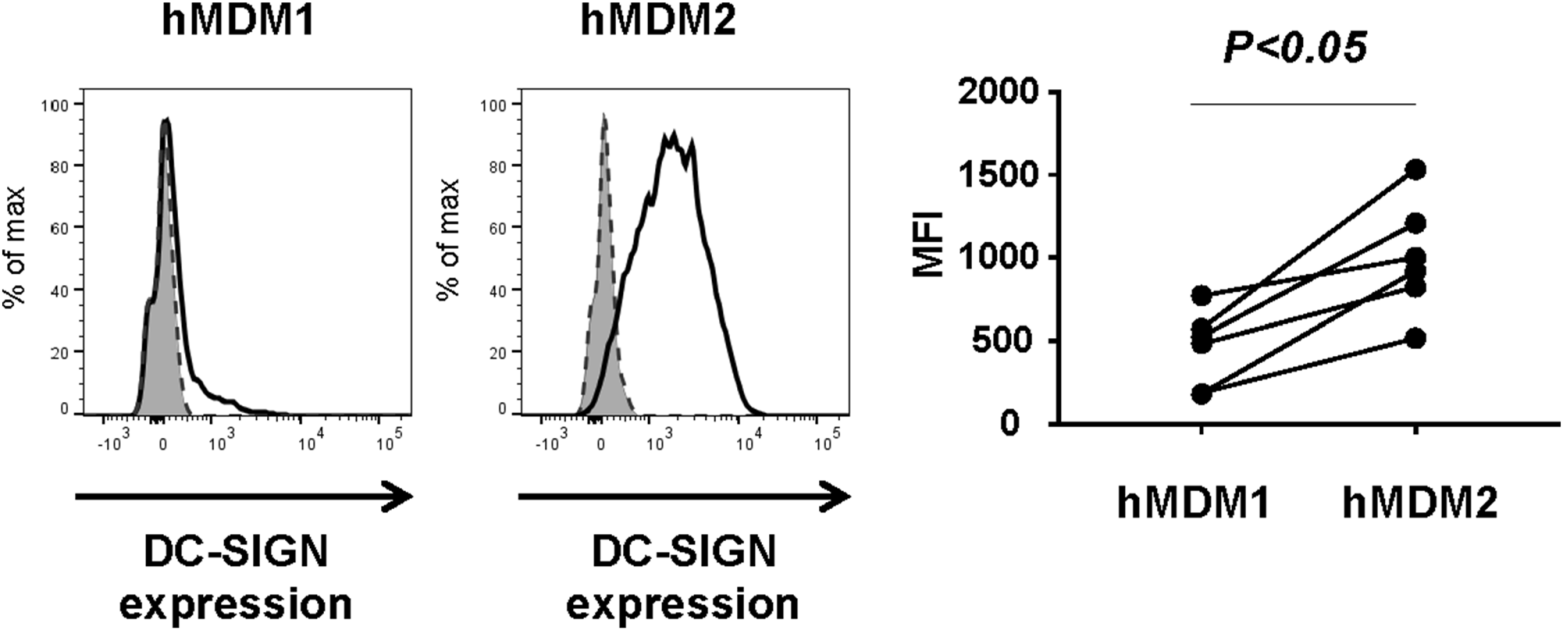
hMDM2, but not hMDM1 expressed DC-SIGN at high levels. hMDM1and hMDM2 were stained with PE-conjugated anti-DC-SIGN mAb. Cell surface expression of DC-SIGN was then measured by FACS. Grey area: cells without the mAb staining, dashed line: cells stained with an isotype control antibody, black line: cells stained with the mAb. Histogram represents three independent experiments using six donors in total. MFI: Mean fluorescence intensity in cells stained with the mAb. Each set of symbols connected by a line represents data from cells of an individual donor.

**Figure 5:**
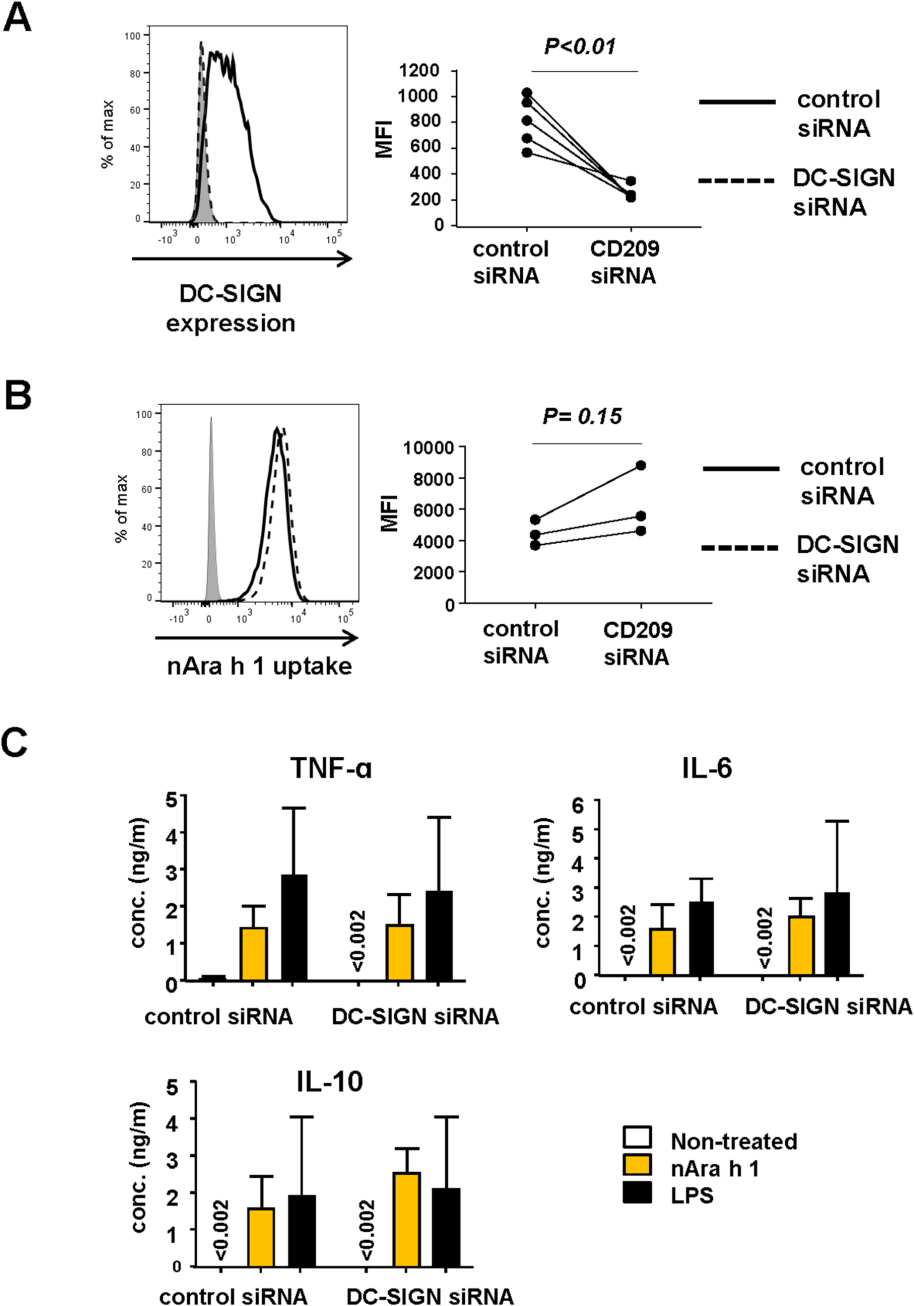
Gene silencing of DC-SIGN does not influence nAra h 1 uptake and activation by hMDM2. hMDM2 were transfected with nontargeting control siRNA or targeting DC-SIGN siRNAs for 96 h. (**A**) The expression of DC-SIGN on the cell surface was measured by FACS. Grey area: cells treated with medium only, solid line: cells treated with control siRNA; dashed line: cells treated with siRNA targeting DC-SIGN. Histogram represents data of three independent experiments using five donors in total. (**B**) After siRNA transfection, the cells were treated with FITC-nAra h 1 for 15 min. The uptake of FITC-nAra h 1 by the cells was measured by FACS. Grey area: cells without any treatment, solid line: cells treated with control siRNA; dashed line: cells treated with siRNA targeting DC-SIGN. Histogram represents two independent experiments using three donors in total. MFI: Mean fluorescence intensity in cells. Each set of symbols connected by a line represents data from cells of an individual donor. (**C**) After siRNA transfection, the cells were incubated in the presence or absence of 50 μg/ml of nAra h 1 (orange bar), or 0.5 μg/ml of LPS (black bar) for 20 h. The concentrations of TNF-ɑ, IL-6, and IL-10 in the cell culture supernatants were measured by ELISA. The data represent three independent experiments using five independent donors in total.

A previous study suggested that DC-SIGN is a receptor of nAra h 1 in its activation of human monocyte-derived DCs^11^. Therefore, we assessed the expression levels of DC-SIGN on the cell surface of hMDM1 and hMDM2. Although nAra h 1 activated hMDM1, the expression of DC-SIGN on hMDM1 was marginal (Fig. 4). In contrast, hMDM2 expressed substantial levels of DC-SIGN. To assess the involvement of DC-SIGN in nAra h 1-induced activation of hMDM2, the cells were transfected with small interfering RNA (siRNA) to knock down the expression of DC-SIGN. hMDM2 showed remarkably reduced expression of DC-SIGN on the cell surface after transfection with siRNA targeting the receptor, whereas the expression was retained on the surface of cells transfected with non-targeting siRNAs (Fig. 5A). Almost no reduction of nAra h 1 uptake was observed in the cells transfected with DC-SIGN siRNAs. In addition, hMDM2 transfected with siRNA targeting DC-SIGN still produced TNF-α, IL-6 and IL-10 in response to Ara h 1, as observed in those transfected with nontargeting siRNA-transfected cells (Fig. 5B). The results suggest that DC-SIGN is dispensable for nAra h 1-induced hMDM2 activation.

**Figure 6:**
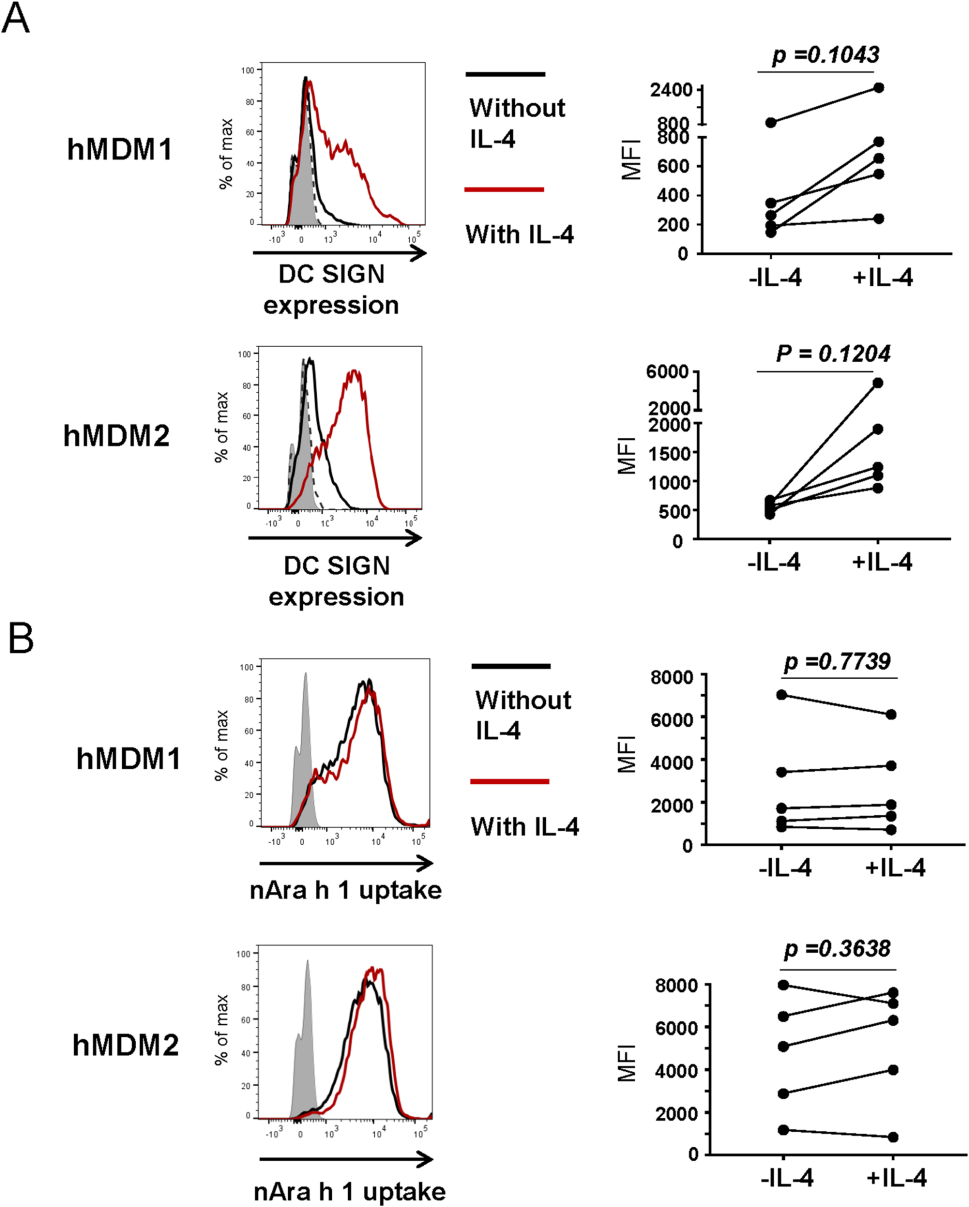
IL-4 enhanced DC-SIGN expression but not nAra h 1 uptake by hMDM1 and hMDM2. hMDM1 and hMDM2 were cultured in the absence or presence of 10 ng/ml of IL-4. (**A**) Expression levels of DC-SIGN were measured by FACS. Grey area: cells without any treatment and staining, dashed line: cells cultured with IL-4 and stained with an isotype control Ab, black line; cells cultured without IL-4 and stained with anti-DC-SIGN mAb, red line: cells cultured with IL-4 and stained with the mAb. (**B**) Uptake levels of FITC-nAra h 1 by the cells were measured by FACS. Grey area: cells without treatment and staining, black line; cells cultured without IL-4 and treated with FITC-nAra h 1, red line: cells cultured with IL-4 and FITC-nAra h 1. Each set of symbols connected by a line represents data from cells of an individual donor. The data represent three independent experiments using five donors in total.

One might argue that DC-SIGN is not expressed sufficiently to bind Ara h 1 on the cell surface of macrophages. Therefore, M1 and M2 macrophages were treated with IL-4. This Th2 cytokine is known to enhance DC-SIGN expression in DC. Indeed, DC-SIGN expression tended to be enhanced in both hMDM1 and hMDM2 after IL-4 treatment (Fig. 6A). However, the levels of nAra h 1 uptake were not altered in IL-4-treated hMDM1 and hMDM2 (Fig. 6B). The results further support that DC-SIGN is not the primary receptor for nAra h 1 in hMDM1 and hMDM2.

### SR-AI, but not DC-SIGN, engages uptake of natural Ara h 1 by hMDM2

**Figure 7:**
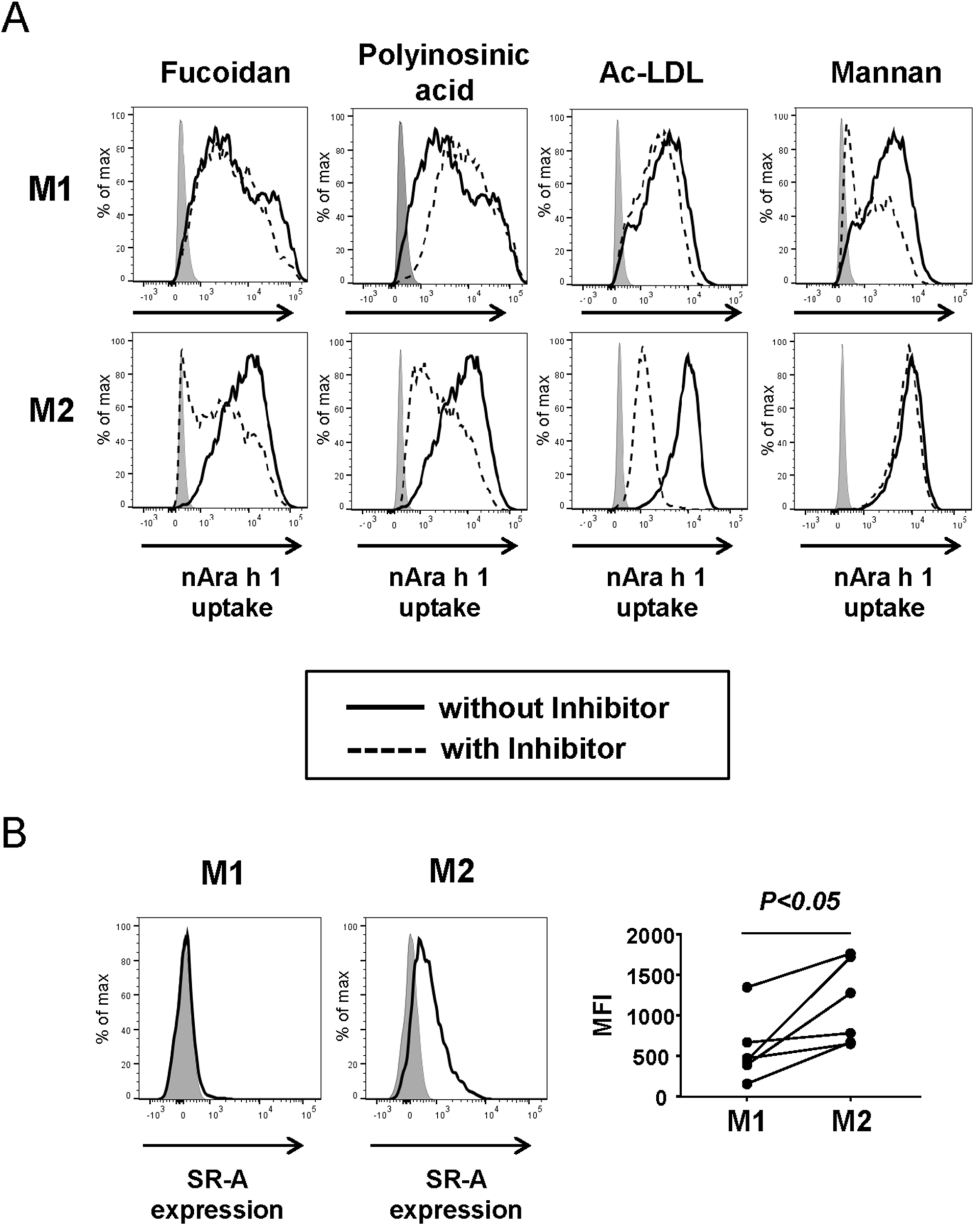
SR-AI inhibitors reduced nAra h 1 uptake by hMDM2. (**A**) hMDM1 and hMDM2 were treated with or without fucoidan, polyinosinic acid, acetylated LDL or Mannan for 30 min prior to the incubation with FITC-nAra h 1. The uptake of Ara h 1 by macrophages was assessed by FACS. Grey area: cells cultured without any treatment, dashed line: cells treated with an inhibitor and FITC-nAra h 1, black line: cells treated without inhibitor and with FITC-nAra h 1. Each set of symbols connected by a line represents data from cells of an individual donor. The data represent three independent experiments using five to seven donors in total. (**B**) Cells were stained with PE-conjugated anti-SR-AI mAb and surface expression of SR-AI measured by FACS. Grey areas represent cells cultured without stimulation. Right-hand side graph represents data from cells of three to six different donors. MFI: Mean fluorescence intensity in cells stained with the anti-SR-AI mAb. Each set of symbols connected by a line represents data from cells of an individual donor.

To identify the responsible receptor(s) for the nAra h 1 uptake by macrophages, we incubated the cells with inhibitors of putative cell surface receptors. Mannan, an inhibitor of DC-SIGN, the mannose receptor (MR), and Dectin-1 reduced nAra h 1 uptake by hMDM1, but not by hMDM2 (Fig. 7A, S8). In contrast, the SR-AI-specific inhibitors acetylated LDL and polyinosinic acid reduced the uptake of nAra h 1 by hMDM2, but not by hMDM1 (Fig. 7A, S8). Fucoidan, an inhibitor of SR-AI, TLR4 and the MR reduced nAra h 1 uptake by hMDM1 and hMDM2. Consistent with this observation, hMDM2 tended to express higher levels of SR-AI in comparison to M1 macrophages (Fig. 7B). Other receptor inhibitors, e.g. SR-B and galectin-3 inhibitors did not reduce nAra h 1 uptake in M1 and M2 macrophages (data not shown). These results suggested that SR-A is a receptor binding to nAra h 1 in hMDM2.

## Discussion

In the present study, we demonstrated that nAra h 1 is capable to activate monocyte-derived human macrophages because of the presence of carbohydrate residues. Interestingly, hMDM1 and hMDM2 recognize nAra h 1 using different receptors. We identified SR-AI as a receptor that recognizes nAra h 1 in hMDM2. SR-AI is trimeric type II transmembrane glycoprotein consisting of a cytoplasmic tail, a transmembrane domain, a spacer region, an α-helical coiled coil domain, a collagenous domain and a terminal cysteine-rich domain with C-type lectin structures^20,27,28^. SR-AI binds to a diverse family of ligands with negatively charge and certain types of carbohydrates that include acetylated-LDL, polyinosinic acid and fucoidan^20,28,29^. These SR-AI ligands inhibited uptake of Ara h 1 by hMDM2 expressing SR-AI. nAra h 1 is heavily glycosylated with high mannose (Man_5_GlcNAc_2_ and Man_6_GlcNAc_2_) and nonfucosylated complex *N-*glycans (Man_4_XylGlcNAc_2_ and Man_3_XylGlcNAc_2_)^18^, which could bind SR-AI.

Shreffler et al. showed that nAra h 1 activates inflammatory responses in hMDDCs by binding to DC-SIGN and thereby skews Th2 differentiation of CD4^+^ T cells^17^. However, we found that interaction of nAra h 1 with hMDM2 was independent from DC-SIGN, because nAra h 1 uptake and allergen-induced cytokine production by the cells were not abolished by siRNA-mediated knockdown of DC-SIGN, or the presence of Mannan, an inhibitor of DC-SIGN. The discrepancy of the observation by Shreffler et al. and ours could be explained by different expression profiles of C-type lectins and SRs in DC and macrophages. Macrophages appear to express receptors to which Ara h 1 preferably binds rather than to DC-SIGN. Indeed, in our experimental setting, the levels of DC-SIGN expression were lower in hMDM1 and hMDM2, compared to hMDDCs (Fig. S9). Notably, nAra h 1 uptake by hMDM1, but not by hMDM2, was inhibited in the presence of Mannan. There are several C-type lectin receptors, e.g., Macrophage inducible C-type “calcium-dependent” lectin (Mincle), DC-associated C-type lectin (Dectin)-1, MR, CD36 and DC-SIGN, which are blocked by mannan^30^ and have the potential to bind high mannose (Man5GlcNAc2 and Man6GlcNAc2) residues in nAra h 1. Among the receptors, MR is highly expressed on the cell surface of hMDM1 (Fig. S8). Bugdorf et al. showed that ligands of MR are transferred into early endosome for cross presentation in APC^31^. However, nAra h 1 was endocytosed but not transferred into early endosomes in hMDM1. The result suggests that MR is not engaged in nAra h 1 uptake by hMDM1. It is also less likely that DC-SIGN is a major receptor to recognize Ara h 1 in hMDM1, since the cells express DC-SIGN only at marginal levels. In addition, IL-4-treated hMDM1 did not enhance uptake of Ara h 1 although IL-4 treatment enhanced expression levels of DC-SIGN in the cells. Further studies are needed to identify receptor(s) binding to Ara h 1 in hMDM1.

Natural Ara h 1 induced predominant production of IL-6 and IL-10 in hMDM1 and hMDM2, respectively, suggesting that this natural form of peanut allergen induces both inflammatory and anti-inflammatory responses, depending on APCs encountering the allergen. A recent study suggested that human M2 macrophages gain a function to produce high levels of IL-10 upon treatment with a therapeutic allergen in allergen-specific immunotherapy (AIT)^32^. In such a condition, nAra h 1 might be a useful therapeutic allergen to promote the efficacy of AIT of peanut allergy.

Evidence has accumulated that the interaction of allergens with epithelial cells, T, B, DC, and macrophages are individually contributing to clinical tolerance and success of AIT^33^. We also found that DC and macrophages are differently activated by natural allergens due to their specific profile of receptor expressions. Sirvent et al. showed that DC-SIGN engagement by therapeutic allergens in DC could promote the efficacy of AIT^34^. However, our results suggest that DC-SIGN is not a primary receptor to influence on macrophage function. The present study underlines the need for understanding mechanisms of allergen-driven inflammation and tolerance by each type of APC.

It is also important to elucidate how SR-AI-mediated inflammatory signals initiated by nAra h 1 impact on Ara h 1-specific CD4^+^ T-cell activation/differentiation and subsequent IgE production. SR-AI is known to trigger inflammatory responses and deliver its ligands into MHC class II loading pathway for enhanced CD4^+^ T-cell activation in APC^28,31^. However, the frequency of food allergen-specific CD4^+^ T-cells is extremely low, lower than those of aero antigen or commensal bacteria specific CD4 positive T-cells in peripheral bloods^35^. To overcome this technical limitation in investigation for food allergen-specific CD4^+^ T-cells, establishment of T-cell lines or clones from food allergic patients would be necessary.

We demonstrated the activation of human macrophages by nAra h 1 isolated from unroasted peanuts and noticed the importance of assessment for stimulatory capacity of nAra h 1 derived from roasted peanuts. Allergic individuals are sensitized by roasted peanuts since peanuts have been roasted prior to delivery to the consumers. Several studies have shown that nAra h 1 undergoes aggregation and is subjected to chemical reactions (e.g., the Maillard reaction) upon thermal processing^36,37^. Therefore, thermal processing could alter the stimulatory capacity of nAra h 1 on APCs. However, aggregated Ara h 1 is insoluble in buffer solutions and cannot be sufficiently isolated from roasted peanuts at high purity that required for cellular analysis.

In summary, we demonstrated the stimulatory capacities of nAra h 1 in hMDM1 and hMDM2. This study is the first to identify that SR-AI acts as receptor for nAra h 1 in human APCs. Our findings advance the knowledge about immunological properties of nAra h 1 and could contribute to develop a novel strategy for treating peanut allergy.

## Supplementary Information

Supplementary Information
