# Supplementary material for: Human monocyte-derived type 1 and 2 macrophages recognize Ara h 1, a major peanut allergen, by different mechanisms
Source: Sci Rep. 2021 May 12;11:10141. doi: 10.1038/s41598-021-89402-1 (PMC8115286; doi:10.1038/s41598-021-89402-1)
Supplement: Supplementary file 1 — Supplementary Information [file 41598_2021_89402_MOESM1_ESM.pdf]

Supporting information

Human monocyte-derived type 1 and 2 macrophages recognize Ara h 1, a major peanut allergen, by different mechanisms

Maren Krause[1], Peter Crauwels[2,3], Frank Blanco[1], Martin Globisch[4], Andrea Wangorsch[1], Thomas Henle[4], Jonas Lidholm[5], Ger van Zandbergen[2,6,7], Stefan Vieths[1], Stephan Scheurer[1], Masako Toda[1,8,*]

[1] VPr1 Research Group "Molecular Allergology", Paul-Ehrlich-Institut, Langen, Germany.

[2] Division of Immunology, Paul-Ehrlich-Institut, Langen, Germany.

[3] Institute of Microbiology and Biotechnology, University of Ulm, Ulm, Germany.

[4] Institute of Food Chemistry, Technical University Dresden, Dresden, Germany.

[5] Thermo Fisher Scientific, Uppsala, Sweden.

[6] Institute for Immunology, University Medical Center, Johannes Gutenberg University Mainz, Mainz, Germany.

[7] Research Center for Immunotherapy (FZI), University Medical Center, Johannes Gutenberg-University Mainz, Mainz, Germany.

[8] Laboratory of Food and Biomolecular Science, Graduate School of Agricultural Science, Tohoku University, Sendai, Japan.

*Corresponding author

Dr. Masako Toda

Laboratory of Food and Biomolecular Science,

Graduate School of Agricultural Science, Tohoku University, Sendai, Japan.

E-mail: masako.toda.a7@tohoku.ac.jp

Abbreviations

Ac-LDL: acetylated low-density lipoprotein

APC: antigen presenting cells

DC: dendritic cells

DC-SIGN: dendritic cell-specific intercellular adhesion molecule-3-grabbing non-integrin

hMDDC: human primary monocyte derived dendritic cells

hMDM1: human primary monocyte derived macrophages type 1

hMDM2: human primary monocyte derived macrophages type 2

LPS: lipopolysaccharides

mAb: monoclonal antibody

nAra h 1: natural Ara h 1

PPRs: pattern recognition receptors

rAra h 1: recombinant Ara h 1

SR-AI: scavenger receptor class A type I

TLR4: Toll-like receptor 4

Materials and Metohds

Generation of human monocyte derived dendritic cells (hMDDC)

Buffy coats obtained from anonymous healthy blood donors were purchased from the German Red Cross Blood Donor Service Baden-Württemberg & Hessen. Human peripheral mononuclear cells were isolated from the buffy coats by passage over a Leukocyte Separation Medium (PAA Laboratories GmbH) gradient. Monocytes were isolated by CD14 positive selection (Miltenyi Biotec GmbH) and differentiated into hMDDC by the addition of 10 ng/mL recombinant human GM-CSF (R&D Systems) and recombinant human IL-4 (Peprotech) for 5 to 7 days[1].

Assessment of receptor expression by FACS

hMDM1, hMDM2 and hMDDC were treated with human Fc block and stained with phycoerythrin (PE)-conjugated anti-human CD36, anti-human SR-AI (CD204), anti-human mannose receptor (CD206), anti-human DC-SIGN (CD209), anti-human mincle, or anti-human Dectin-1 mAb. Antibodies used for this experiment were purchased from Thermo Fisher Scientific.

Binding analysis of nAra h 1 to Toll-like receptor 4 (TLR4)

HEK-Dual™ human TLR4 (NF/IL8) cells (InvivoGen) were stimulated with various concentrations of nAra h 1 or LPS for 20 hours. The concentrations of IL-8 in the culture supernatant were measured by ELISA (Peprotech) as an indication of TLR4 engagement.

Reference

1. Filippis, C., Arens, K., Noubissi Nzeteu, GA., et al., Anti-Tumor Necrosis Factor α Therapeutics Differentially Affect Leishmania Infection of Human Macrophages. *Front. Immunol.* ***8**, 1880*. https:// doi: 10.3389/fimmu.2017.01880. (2017)

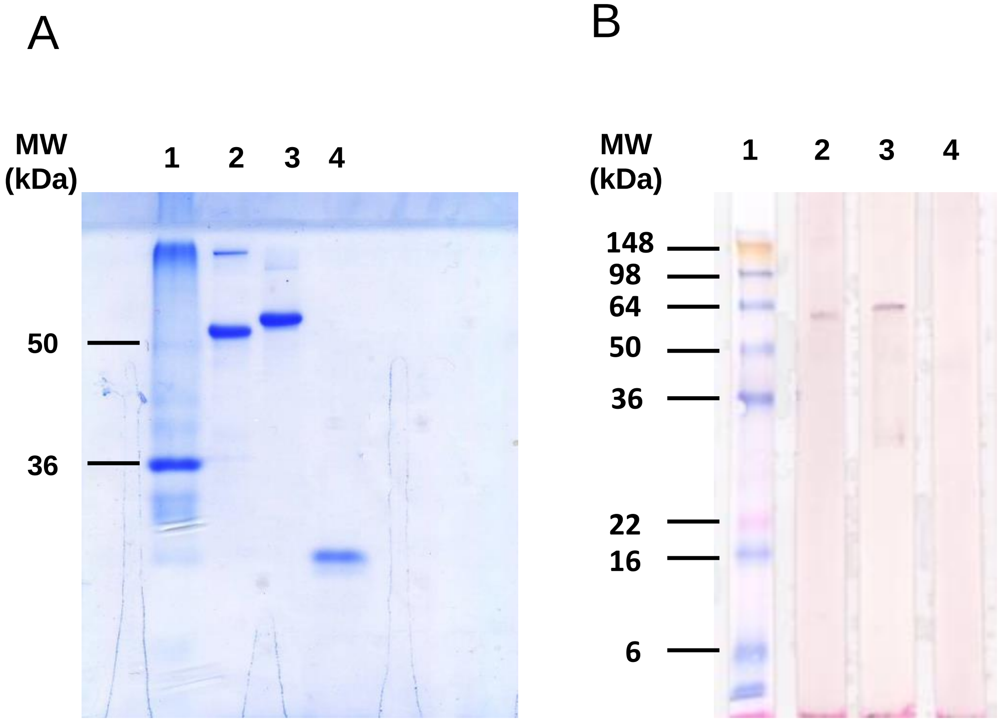

Lane 1 Size marker
Lane 2 nAra h 1
Lane 3 rAra h 1
Lane 4 rAra h 2

Lane 1 Size marker
Lane 2 nAra h 1
Lane 3 rAra h 1
Lane 4 Secondary antibody control

Fig. S1: Analysis of natural and recombinant Ara h 1 by SDS-PAGE and Immunoblotting. (A) SDS-PAGE and Coomassie staining of purified nAra h 1 from peanut, rAra h 1 and rAra h 2. (B) nAra h 1 and rAra h 1 blotted onto nitrocellulose membrane detected using mouse anti-Ara h 1 monoclonal antibody, followed by alkaline phosphatase-conjugated anti-mouse IgG antibodies, MW, Molecular weight marker (kDa).

Fig. S1

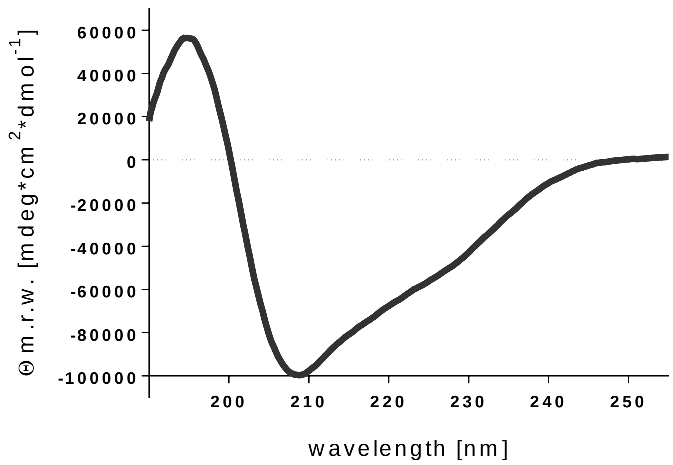

Fig. S2: CD spectra of recombinant Ara h 1. CD spectroscopy of rAra h 1 was performed on a J-810S spectropolarimeter (Jasco) with constant nitrogen flushing at 20° C.

Fig. S2

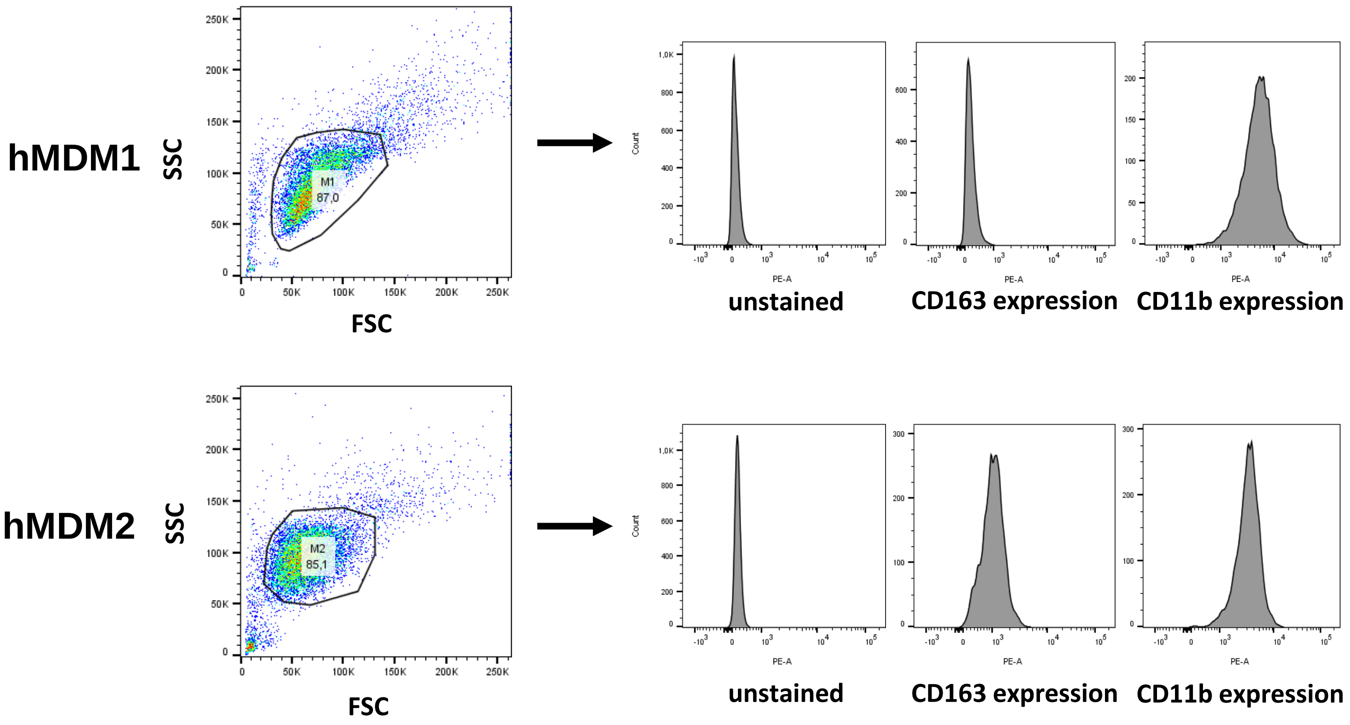

Fig. S3: Gating strategy in FACS analysis. The main population of hMDM1 and hMDM2 is CD11b^{+}CD163^{-} and CD11b^{+}CD163^{+} cells, respectively.

Fig. S3

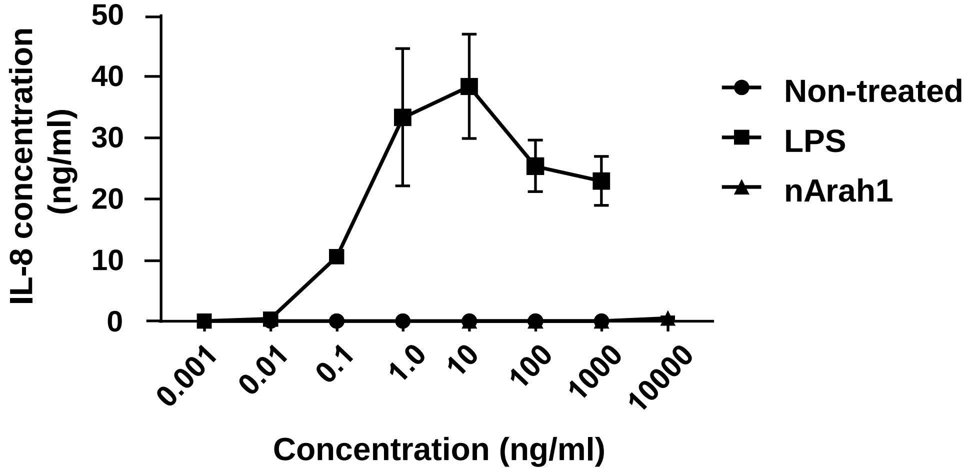

Fig. S4: nAra h 1 did not bind TLR4. HEK-Dual™ human TLR4 (NF/IL8) cells were stimulated with 0.001-10000 ng/mL of nAra h 1 or LPS for 20 hours. The concentrations of IL-8 in the culture supernatant were measured by ELISA. The data are representative of two experiments.

Fig. S4

hMDM1

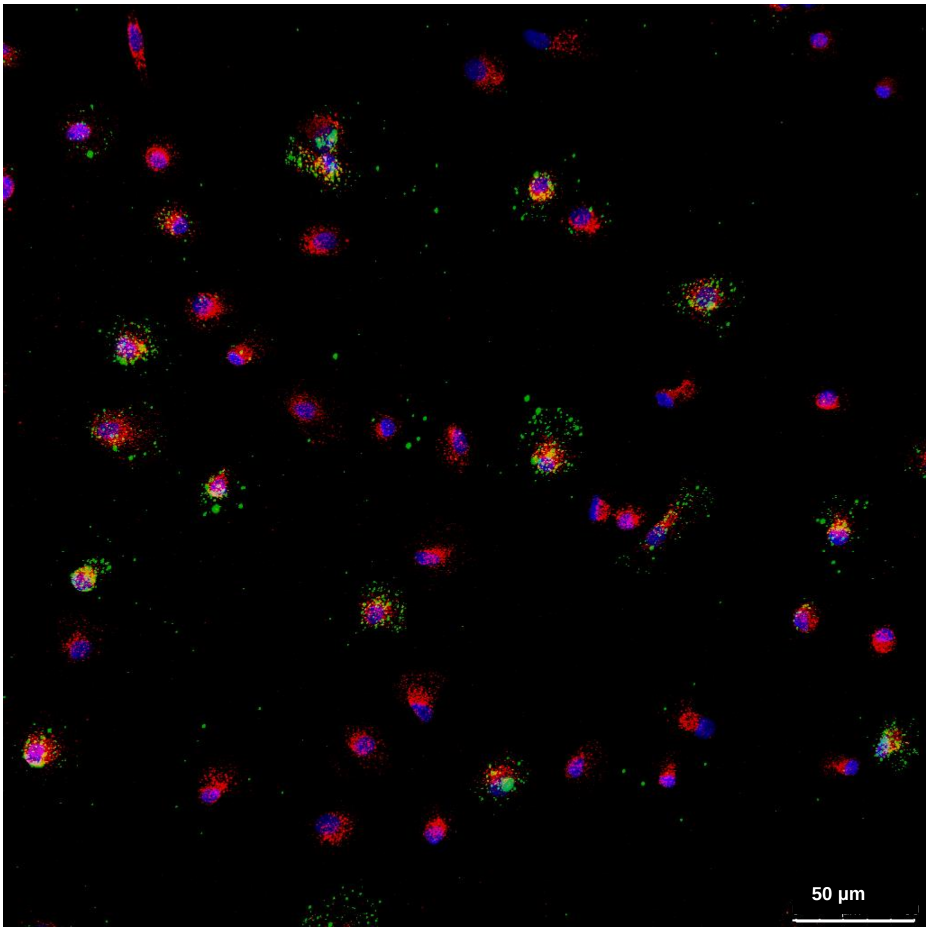

Green: FITC-nAra h 1   Red: EEA1   Blue: DAPI

Fig. S5: Uptake of FITC-nAra h 1 by hMDM1 in confocal imaging. hMDM1 were incubated with 10 µg/ml of FITC-nAra h 1 for 15 min. The cells were then blocked with normal goat serum, stained with anti-human EEA1 in combination with Cy3-conjugated goat anti-mouse IgG, F(ab')2 fragment, and treated with 4′,6-diamidino-2-phenylindole. The cell images was obtained by confocal microscopy. Data is representative of two independent experiments using four donors in total.

Fig. S5

hMDM2

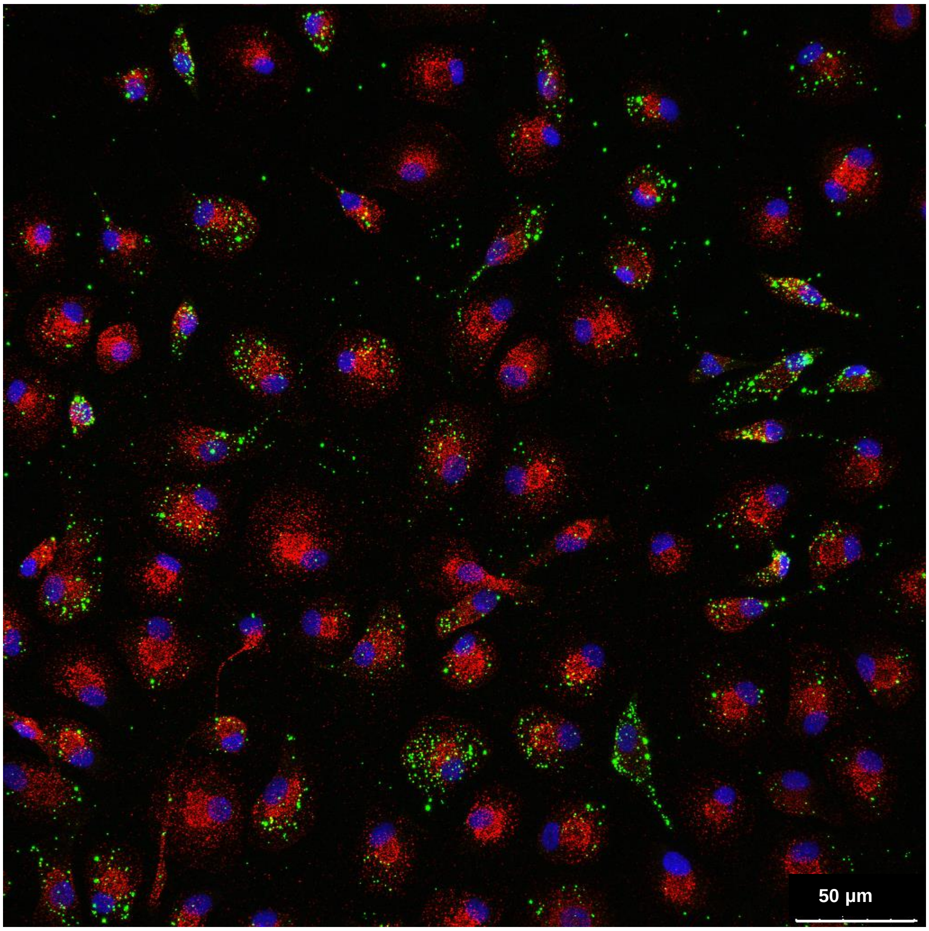

Green: FITC-nAra h 1 Red: EEA1 Blue: DAPI

Fig. S6: Uptake of FITC-nAra h 1 by hMDM2 in confocal imaging. hMDM2 were incubated with 10 µg/ml of FITC-nAra h 1 for 15 min. The cells were then blocked with normal goat serum, stained with anti-human EEA1 in combination with Cy3-conjugated goat anti-mouse IgG, F(ab')2 fragment, and treated with 4′,6-diamidino-2-phenylindole. The cell images was obtained by confocal microscopy. Data is representative of two independent experiments using four donors in total.

Fig. S6

A

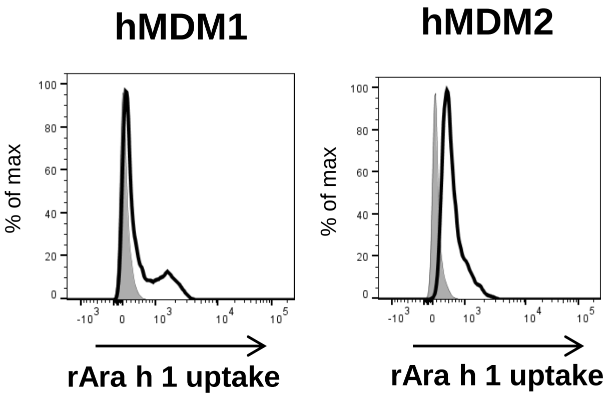

Fig. S7: Recombinant Ara h 1 was not taken up by macrophages. hMDM1 and hMDM2 were incubated with 5.0 µg/ml of FITC-conjugated recombinant Ara h 1 (FITC-rAra h 1) for 15 min. The uptake levels of FITC-rAra h 1 by the cells were measured by FACS. Grey areas represent cells cultured without rAra h 1 incubation. The data are representative of two independent experiments using three donors in total.

Fig. S7

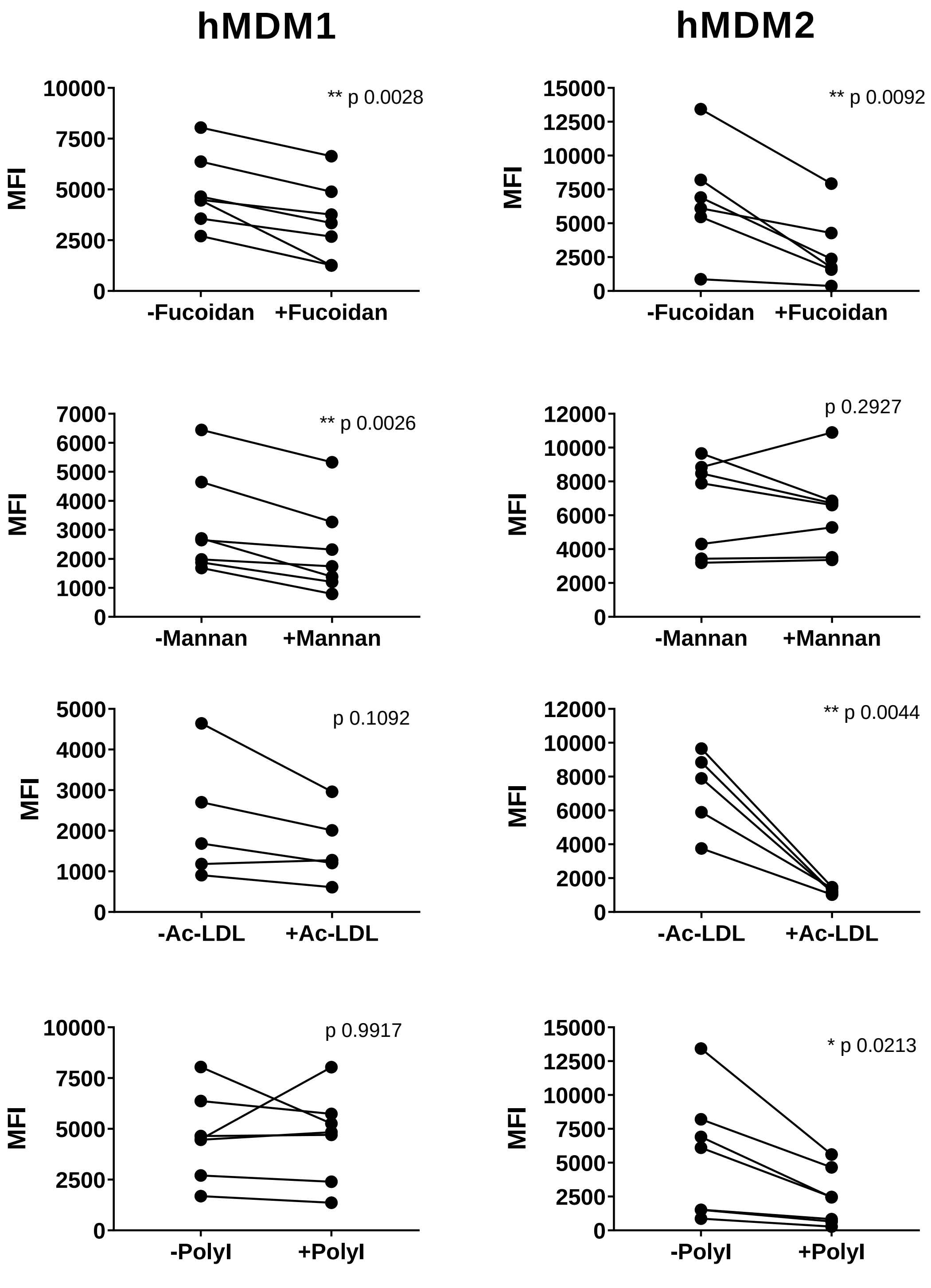

Fig. S8: SR-AI inhibitor suppressed uptake of nAra h 1 by macrophages. hMDM1M1 and hMDM2M2 were treated with or without 100 μg/ml of fucoidan, or 10 μg/ml of polyinosinic acid, 200 μg/ml of Acetylated LDL or 200 μg/ml of Mannan for 30 min prior to the incubation with 10 μg/ml of FITC-nAra h 1. The uptake of Ara h 1 by macrophages was assessed by FACS. Each set of symbols connected by a line represents for data from cells of an individual donor. The data were collected from three independent experiments using five to seven donors in total.

Fig. S8

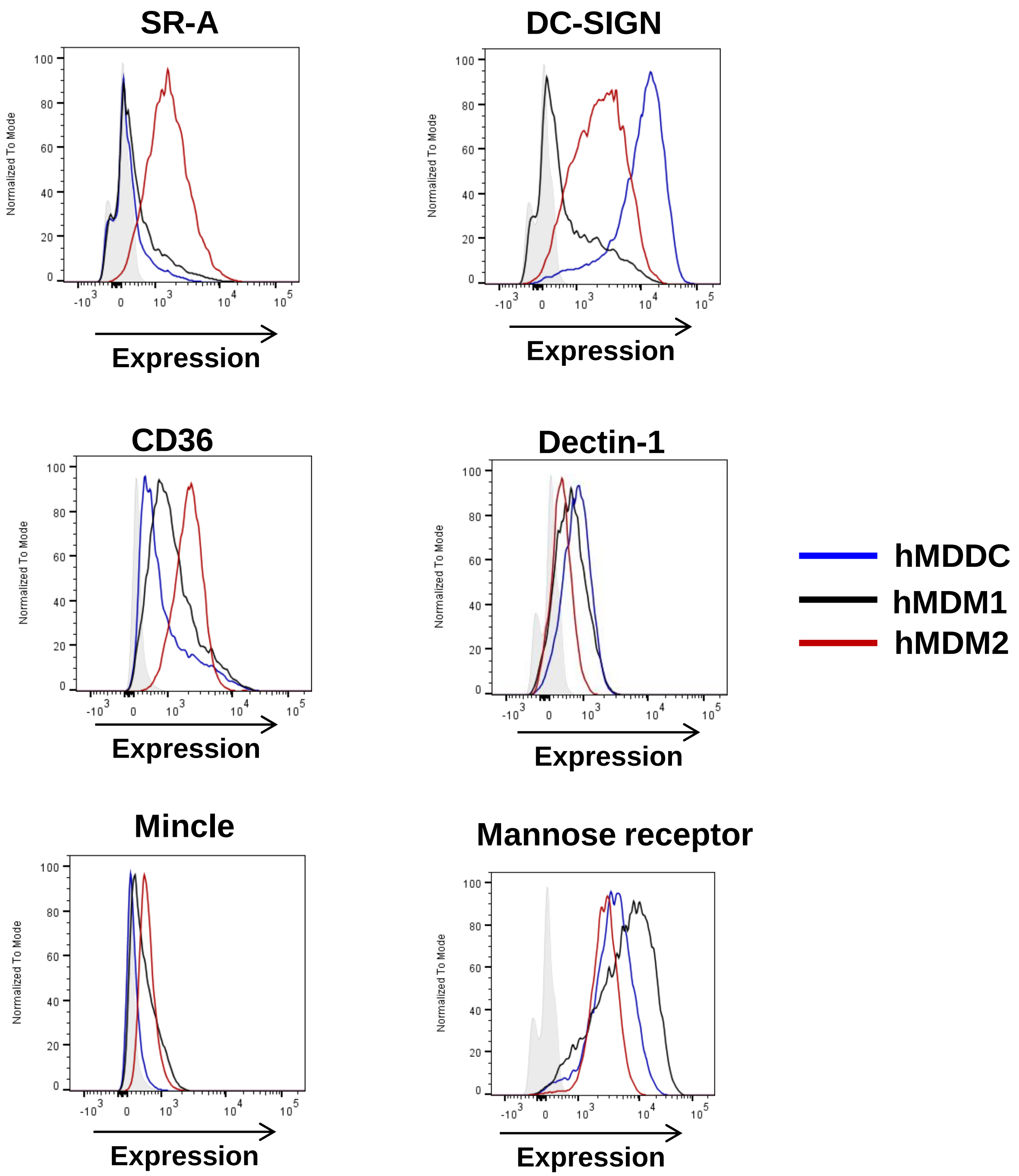

Figure S9: Receptor expression in hMDDC, hMDM1 and hMDM2. The expression levels of receptors on the cell surface of hMDDC, hMDM1 and hMDM2 were assessed by FACS. The gray area represent non-stained cells. The figure shows data of cells generated from one doner. The experiment repeated twice using two doner in total.

Fig. S9
